# Gastroesophageal reflux disease in an area with low *Helicobacter pylori* infection prevalence

**DOI:** 10.1371/journal.pone.0205644

**Published:** 2018-11-14

**Authors:** Muhammad Miftahussurur, Dalla Doohan, Iswan Abbas Nusi, Pangestu Adi, Yudith Annisa Ayu Rezkitha, Langgeng Agung Waskito, Kartika Afrida Fauzia, Taufan Bramantoro, Ummi Maimunah, Husin Thamrin, Safitri Indah Masithah, Sukadiono Sukadiono, Tomohisa Uchida, Maria Inge Lusida, Yoshio Yamaoka

**Affiliations:** 1 Gastroentero-Hepatology Division, Department of Internal Medicine, Faculty of Medicine-Dr. Soetomo Teaching Hospital, Universitas Airlangga, Surabaya, Indonesia; 2 Department of Environmental and Preventive Medicine, Oita University Faculty of Medicine, Yufu, Japan; 3 Institute of Tropical Disease, Universitas Airlangga, Surabaya, Indonesia; 4 Muhammadiyah University of Surabaya, Surabaya, Indonesia; 5 Department of Dental Public Health, Faculty of Dental Medicine, Universitas Airlangga, Surabaya, Indonesia; 6 Department of Molecular Pathology, Oita University Faculty of Medicine, Yufu, Japan; 7 Department of Gastroenterology and Hepatology, Baylor College of Medicine and Michael E. DeBakey Veterans Affairs Medical Center, Houston, Texas, United States of America; National Cancer Center, JAPAN

## Abstract

The association between gastroesophageal reflux disease (GERD) prevalence and its risk factors in an area with low *Helicobacter pylori* prevalence is important to clarify. We analyzed the prevalence of GERD and risk factors in an area of Indonesia with low prevalence of *H*. *pylori* infection. We recruited 104 dyspeptic patients who underwent endoscopy in Surabaya. Patients were diagnosed with GERD based on the Los Angeles classification. We evaluated gastric biopsy specimens and measured serum pepsinogen levels. Interleukin polymorphisms were evaluated by polymerase chain reaction-restriction fragment length polymorphism. Of 104 patients, 56 (53.8%) were endoscopically found to have GERD, with most categorized as grade A; 48 (46.2%) were classified as non-GERD. Higher economic status, smoking, and a history of proton-pump inhibitor use significantly increased the risk of GERD. GERD Questionnaire scores showed a positive correlation with GERD (*P* < 0.001). An association was found between antral atrophic gastritis and GERD (*P* = 0.030), and patients with GERD more frequently had severe antral atrophy than nonerosive reflux disease (*P* = 0.018). We found an association between pepsinogen I/II levels and GERD (*P* = 0.047), but with low accuracy. IL-1β -511 TT and CT were predominant among the IL-1β -511 genotypes, and IL-8–251 AT and TT were predominant among the IL-8–251 genotypes. In conclusion, we found a high prevalence of GERD in an area with low prevalence of *H*. *pylori* infection, which could be associated with acid reflux. Smoking, history of proton-pump inhibitor use, and higher economic group significantly increased the risk of GERD.

## Introduction

*Helicobacter pylori* infection, the most prevalent human chronic bacterial infection [1], modifies gastric acid secretion, affecting gastroduodenal disease pathogenesis, including gastroesophageal reflux disease (GERD) [2], GERD is a condition wherein gastric reflux or complications expose the gastric contents to the esophageal squamous epithelium [3].

The hypothesis regarding an *H*. *pylori*–GERD association has been reinforced by the parallel of increasing GERD incidence with decreasing *H*. *pylori* infection prevalence in Asia [4, 5]. However, some authors consider GERD as an acid reflux-related disease and *H*. *pylori* as a biological secretory or anti-secretory agent [6, 7]. Acid secretion in corpus predominant gastritis decreases, thus inhibiting severe reflux development, contrary to antral predominant gastritis. A meta-analysis was also unable to prove a significant association between *H*. *pylori* eradication and GERD development [8]. The prevalence and risk factors for GERD in an area of low *H*. *pylori* prevalence must be examined to provide more information regarding GERD–*H*. *pylori* association.

Reflux disease is classified into GERD and non-erosive reflux disease (NERD) [9]. GERD is diagnosed based on the presence of mucosal breaks or ulcerations found during an endoscopic examination. NERD is defined as the presence of reflux-related symptoms in the absence of esophageal mucosal breaks or ulcerations during endoscopy examination [9]. Intraesophageal pH monitoring also facilitates GERD diagnoses in the absence of endoscopically visible lesions. These methods are less comfortable for patients and less feasible in some areas. Indonesia has a population of more than 255.5 million people in 2015 living on thousands of islands (Statistics Indonesia, http://www.bps.go.id/); however, only 313 hospitals have gastrointestinal endoscopy systems, and most are located on the main island, Java [10]. Additionally, only two centers have intraesophageal pH monitoring to confirm GERD diagnoses (Dr. Miftahussurur, personal communication). Thus, an indirect method of diagnosing GERD, such as the GERD Questionnaire (GERDQ), is one option, which has recently been validated in several countries and is reportedly a useful complementary diagnosis tool for GERD in primary care [11–13].

Several lifestyle factors, such as smoking [14, 15], table salt consumption [15], obesity [16, 17], older age [14], irregular diet, and diet variety [18] have been established as risk factors for GERD. Inflammation mediators may play a more important role in GERD pathogenesis than caustic acid injury [19]. Interleukin (IL)-1β, an important proinflammatory cytokine that increases in the mucosal tissue in esophagitis and Barrett’s esophagus [20], could be correlated with decreased esophageal contraction, which is caused by acetylcholine release inhibition from neurons [21]. Research on Taiwanese patients found that IL-1β polymorphisms affect gastritis and erosive reflux esophagitis [22]. IL-8 mediated chemotaxis in neutrophils is found to be involved in both the acute and chronic inflammation processes [23]. A significant association has been shown between IL-8 mRNA in esophageal mucosa and reflux esophagitis based on the Los Angeles classification, and a higher IL-8 mRNA level has been detected in patients with GERD compared with those with a noninflamed esophagus [20, 24].

To our knowledge, reports investigating GERD in Indonesia are scarce [25, 26], and no study has analyzed the risk factors for GERD considering *H*. *pylori* infection status. This study investigated GERD prevalence in areas with low rates of *H*. *pylori* infection and risk factors for GERD.

## Materials and methods

### Study participants

A total of 113 patients with dyspeptic symptoms (e.g. epigastric pain, heart burn, and regurgitation) underwent upper endoscopy between October 2014 and November 2015. We excluded a total 9 patients including 3 patients due to partial gastric resection history, and also two and four patients associated with antibiotic and proton-pump inhibitor (PPI) consumptions, respectively. Finally, 104 participants which were predominantly Javanese included in this study. During the 2 weeks before the endoscopy, these patients had not taken any nonsteroidal anti-inflammation drug (NSAID), PPI, antibiotic, and histamine receptor antagonist. On the day of the endoscopy, we collected the patient's fasting serum and stored it at −20 °C. Sociodemographic data were collected during the interview.

Ethical approval was obtained from the Ethics Committee of Dr. Soetomo Teaching Hospital (Surabaya, Indonesia) and Oita University Faculty of Medicine (Yufu, Japan). Prior to the data collection, the study was explained to the patients, who then provided a written informed consent document based on the guidelines of the Declaration of Helsinki.

### Endoscopic evaluation and GERD Questionnaire

GERD was diagnosed by endoscopy. To evaluate the severity of reflux esophagitis, an endoscopic examination was performed on all the recruited patients by two experienced endoscopists (PA and IAN). During each patient's endoscopy session, we collected three gastric biopsy specimens, two of which was taken from the lesser curvature of the antrum approximately 3 cm from the pyloric ring, and the other from the greater curvature of the corpus (8–10 cm from the esophagogastric junction). One of homogenized antral biopsy specimens was inoculated onto selective agar plates and incubating the plates up to ten days in microaerophilic environment (10% O_2_, 5% CO_2_, and 85% N_2_) at 37°C to isolate *H*. *pylori* as previously described [27]. Two other gastric biopsies were used for histology examination. The esophagitis evaluation was based on the Los Angeles classification [28]. Grade A represents one or more mucosal breaks confined to mucosal folds (not more than 5 mm in length for each); grade B represents at least one mucosal break greater than 5 mm; grade C represents a continuous mucosal break between the tops of two mucosal folds but not circumferential; and grade D is a circumferential mucosal break.

Before undergoing endoscopy, the patients were asked to complete the GERDQ, which is a six-item questionnaire used as an indirect method to help diagnose GERD [29]. The items include questions regarding symptoms of heartburn, regurgitation, epigastric pain, nausea, difficulty getting a good night sleep, and the frequency of taking reflux symptom medication during the previous 7 days. The frequency of positive predictors of GERD was determined using the four-grade Likert scale (0–3) and the negative predictors of GERD by the reversed Likert scale (3–0), resulting a total possible score from 0 to 18. The positive predictors are the consumption of over-the-counter medication for reflux symptoms, regurgitation, heartburn, and sleep disturbance, whereas the two negative predictors are nausea and epigastric pain [12]. We classified NERD as a condition when reflux-related symptoms are present in the absence of esophageal mucosal breaks.

### Histology, *H*. *pylori* status, and pepsinogen

We fixed biopsy materials in 10% formalin and embedded them in paraffin. May–Grünwald–Giemsa stain along with hematoxylin eosin stain was applied to the thin slices of paraffin-embedded biopsy. On the basis of the updated Sydney system, an experienced pathologist (TU) assessed the degree of inflammation, atrophy, and bacterial density in each specimen and assigned to each one of four grades: 0, normal; 1, mild; 2, moderate; and 3, marked [30]. We also assessed the stage of gastritis based on the Operative Link for Gastritis Assessment (OLGA) system [31, 32]. We performed immunohistochemistry with anti-*H*. *pylori* antibody, and *H*. *pylori*-positive cases were regarded as the specimens that had bacterial loads greater than or equal to grade 1 [30]. *H*. *pylori* infection diagnosis was concluded by the combined result of three different tests: histology, culture, and immunohistochemistry. *H*. *pylori*-positive cases were confirmed if at least one positive result was shown in these tests.

Pepsinogen (PG) I and II levels were assessed in the collected serum using a PG ELISA (Eiken, Tokyo, Japan), following the manufacturer's instructions.

### Genotyping for interleukin polymorphisms

For DNA isolation, 100 μL of gastric homogenates was used and extracted using the phenol–chloroform method. IL-1β and IL-8 polymorphisms were evaluated by polymerase chain reaction-restriction fragment length polymorphism (PCR-RFLP) analysis. The primer sequence and PCR condition are shown in Table 1. For genotyping of the IL-1β -511 polymorphism, 10 μL PCR products were digested with 3U *AvaI* (New England Biolabs Japan) and then incubated at 37 °C for 3 h, resulting in 190 and 114 bp (-511 CC); 304, 190, and 114 bp (-511 CT); or they remained intact (-511 TT) [33]. For genotyping of the IL-8–251 polymorphism, 10 μL PCR products were digested with 3U of *MfeI* (New England Biolabs Japan) and then incubated at 37 °C for 1 h, which resulted in 449 and 92 bp (-251 AA); 541, 449, and 92 bp (-251 AT); or they remained intact (-251 TT) [34]. The digested product was confirmed by 2% agarose gel electrophoresis.

**Table 1:** Primer sequence and PCR condition.

Gene	Primer Sequence	Amplification Cycles
IL-1β -511		94 °C for 5 min, then 35 cycles of 94 °C for 1 min, 55 °C for 1 min, 72 °C for 1 min, and 72 °C for 7 min
Forward	5′-TGGCATTGATCTGGTTCATC-3′	
Reverse	5′-GTTTAGGAATCTTCCCACTT-3′	
IL-8–251		95 °C for 3 min, then 35 cycles at 95 °C for 30 s, 54 °C for 1 min, 72 °C for 1 min, and 72 °C for 7 min
Forward	5′-TCATCCATGATCTTGTTCTAA-3′	
Reverse	5′-GGAAAACGCTGTAGGTCAGA-3′	

### Statistical analysis

The statistical analysis was performed using the SPSS statistical software package version 18.0 (SPSS, Inc., Chicago, IL, USA). The categorical data were analyzed using the chi-squared test or Fisher's exact test. Statistical significance was determined when *P* was less than 0.05. The expected genotype frequencies and observed genotype frequencies were calculated using the Hardy–Weinberg Equilibrium equation (*p*^2^ + 2*pq* + *q*^2^ = 1) and tested for compliance using the chi-squared test. Binary and multiple logistic regression were used to analyze the odds ratio (OR) with 95% confidence interval (CI) of the risk factors that could have an association with the development of GERD. The Hosmer–Lemeshow goodness-of-fit test was used to evaluate the fit of the model.

## Results

### GERD prevalence and risk factors

Of 104 patients, 56 (53.8%) were found by endoscopy to have GERD, and 48 patients (46.2%) were classified as non-GERD. According to the LA Classification, 50 were categorized as grade A (89.3%), 4 were categorized as grade B (7.1%), and only two patients were categorized as grade C (3.6%). Forty-eight patients without esophagitis were regarded as non-GERD.

Table 2 shows the adjusted OR calculated for the occurrence of GERD, which used the lowest prevalence of GERD as the reference. The mean age of the patients was 46.42 ± 13.83 years (range, 17–77 years). The young (<30 years of age) had the highest risk of GERD, although it was not significant (OR = 2.57, *P* = 0.148). Of the male patients, 28 (28/44; 63.6%) had GERD, and they showed a higher risk tendency than those in the female group (28/60, 46.7%, *P* = 0.088). The prevalence of GERD in the patients with obesity and overweight (13/21; 61.9% and 7/10; 70.0%, respectively) was higher than in those who were normal and underweight (31/60; 51.7% and 5/13; 38.5%). However, the distribution of BMI in the GERD and non-GERD groups showed no significant association (*P* = 0.391). The high and middle economic groups based on monthly income (1 USD = 13.500 rupiah) had 4.08 and 3.08 times the risk of GERD, respectively, than the low economic group (*P* = 0.021 and *P* = 0.046, respectively). There was an association between obesity and economic status (*P* = 0.045, *r* = 0.197). Smoking was shown to be an important factor in GERD development, given that more than three-quarters of the patients with a history of smoking cigarettes had GERD (19/25; 76.0%) and one-third of the patients with GERD had a history of smoking (19/56; 33.9%). Statistical analyses also showed that the smokers had a significantly higher risk of developing GERD compared with nonsmokers (OR = 3.60, *P* = 0.014). The history of PPI usage also positively increased the risk of GERD, by 2.52 times (*P* = 0.027). After adjusting for age and sex, it was found that high and middle economic status, smoking habits, and the history of PPI use significantly increased the risk of GERD (OR = 8.49, OR = 6.45, OR = 3.22, and OR = 3.12, *P* < 0.05, respectively). However, in the multivariate analysis using logistic regression, only high economic status and PPI use were independent risk factors for GERD. There was no significant association between GERD and ethnicity, religion, marital status, alcohol consumption, antibiotic, or NSAID intake, diabetes mellitus, and hypertension (*P* > 0.05). When we diagnosed *H*. *pylori* infection based on the combination of culture and histology-immunohistochemistry, we only detected *H*. *pylori* as positive in two patients (2/104; 1.9%), which was similar in prevalence to the previous study [27, 35]. Thus, we could not measure their association with GERD due to an insufficient number of cases to analyze. However, all *H*. *pylori*-infected patients were GERD positive; one without atrophy and one with antral atrophy in the histological analysis.

**Table 2:** Association of demographic data and interleukin polymorphism with GERD status.

Variables	Total GERD (%)	Total Non-GERD (%)	Crude OR	OR (95% CI)	*P* Value
**Sex**					
Male	28 (63.6)	16 (36.4)	2.00	0.902–4.436	0.088
Female	28 (46.7)	32 (53.3)	1.00		
**Age**					
<30	10 (66.7)	5 (33.3)	2.57	0.714–9.255	0.148
31–40	10 (50.0)	10 (50.0)	1.29	0.419–3.944	0.660
41–50	12 (57.1)	9 (42.9)	1.71	0.564–5.208	0.342
>60	10 (62.5)	6 (37.5)	2.14	0.627–7.329	0.224
51–60	14 (43.8)	18 (56.3)	1.00		
**BMI**					
<18.5	5 (38.5)	8 (61.5)	1.00		
18.5–24.9	31 (51.7)	29 (48.3)	1.71	0.502–5.832	0.391
25–29.9	13 (61.9)	8 (38.1)	2.60	0.627–10.786	0.188
>30	7 (70.0)	3 (30.0)	3.73	0.646–21.577	0.141
**Economic**					
< Rp1,5M	6 (28.6)	15 (71.4)	1.00		
Rp 1.5M–Rp 5M	29 (58.0)	21 (42.0)	3.08	1.018–9.289	0.046*
> Rp 5M	21 (63.6)	12 (36.4)	4.08	1.241–13.431	0.021*
**Smoking**					
Yes	19 (76.0)	6 (24.0)	3.60	1.298–9.955	0.014*
No	37 (46.8)	42 (53.2)	1.00		
**Alcohol**					
Yes	6 (85.7)	1 (14.3)	5.64	0.654–48.619	0.115
No	50 (51.6)	47 (48.5)	1.00		
**Diabetes**					
Yes	6 (75.0)	2 (25.0)	2.76	0.530–14.366	0.228
No	50 (52.1)	46 (47.9)	1.00		
**Hypertension**					
Yes	14 (60.9)	9 (39.1)	1.44	0.562–3.713	0.445
No	42 (51.9)	39 (48.2)	1.00		
**Ethnicity**					
Java	38 (57.6)	28 (42.4)	1.81	0.740–4.422	0.193
Other	4 (66.7)	2 (33.3)	2.67	0.417–17.046	0.300
Dayak	2 (50.0)	2 (50.0)	1.33	0.164–10.867	0.788
Chinese	12 (42.9)	16 (57.1)	1.00		
**Religion**					
Islam	43 (60.6)	28 (39.4)	4.61	0.868–24.464	0.073
Christian	11 (55.0)	9 (45.0)	3.67	0.590–22.783	0.163
Catholic	2 (25.0)	6 (75.0)	1.00		
Other	0 (0.0)	5 (100)			
**Marital Status**					
Married	48 (54.6)	40 (45.5)	1.20	0.413–3.485	0.737
Not married	8 (50.0)	8 (50.0)	1.00		
**Toilet**					
Toilet	55 (53.4)	48 (46.6)			
Non-toilet	1 (100)	0 (0.0)			
**PPI**					
Yes	41 (62.1)	25 (37.9)	2.52	1.109–5.703	0.027*
No	15 (39.5)	23 (60.5)	1.00		
**Antibiotic**					
Yes	11 (55.0)	9 (45.0)	1.60	0.398–2.822	0.908
No	45 (53.6)	39 (46.4)	1.00		
**NSAID**					
Yes	15 (62.5)	9 (37.5)	1.59	0.622–4.040	0.334
No	41 (51.3)	39 (48.8)	1.00		
**Sambal Intake**					
Yes	28 (56.0)	22 (44.0)	1.18	0.546–2.559	0.672
No	28 (51.9)	26 (48.2)	1.00		
***H*. *pylori* infection**					
Positive	2 (100)	0 (0.0)			
Negative	54 (52.9)	48 (47.1)			
**Antrum Atrophy**					
Yes	11 (84.6)	2 (15.4)	0.18	0.037–0.848	0.030*
No	45 (49.5)	46 (50.6)	1.00		
**Corpus Atrophy**					
Yes	1 (50.0)	1 (50.0)	1.17	0.071–19.225	0.912
No	55 (53.9)	47 (46.1)	1.00		
**OLGA Score**					
Normal	44 (49.4)	45 (50.6)	1.00		
Stage 1	12 (80.0)	3 (20.0)	4.09	1.080–15.493	0.038*
Stage 2	0 (0.0)	0 (0.0)			
Stage 3	0 (0.0)	0 (0.0)			
Stage 4	0 (0.0)	0 (0.0)			
**IL-1β Polymorphism**					
TT	21 (48.8)	22 (51.2)	1.00		
CC	14 (56.0)	11 (44.0)	1.33	0.495–3.590	0.569
CT	21 (58.3)	15 (41.7)	1.47	0.601–3.580	0.400
**T Carrier IL-1β**					
T carrier	32 (46.4)	37 (53.6)	1.00		
CC	24 (68.6)	11 (31.4)	1.12	0.454–2.771	0.804
**C Carrier IL-1β**					
TT	21 (48.8)	22 (51.2)	1.00		
C carrier	35 (57.4)	26 (42.6)	1.41	0.644–3.090	0.390
**IL-8 Polymorphism**					
TT	18 (48.7)	19 (51.4)	1.35	0.424–4.323	0.610
AT	31 (62.0)	19 (28.0)	2.33	0.759–7.158	0.139
AA	7 (41.2)	10 (58.8)	1.00		
**T Carrier IL-8**					
T carrier	49 (60.5)	38 (39.5)	1.84	0.642–5.289	0.256
AA	7 (41.2)	10 (58.8)	1.00		
**A Carrier IL-8**					
TT	18 (48.7)	19 (51.4)	1.00		
A carrier	38 (56.7)	29 (43.3)	1.38	0.618–3.096	0.430

*Statistically significant when *P* < 0.05.

### Endoscopic findings and GERDQ

Most of the patients had low GERDQ scores; for example, 69.2% (72/104) had scores ≤ 3. When we compared the occurrence of the symptoms listed in the GERDQ, as we had expected, heartburn, regurgitation, epigastric pain, nausea, and sleep disorder symptoms were significantly higher in the patients with GERD than in those without (*P* = 0.008, *P* = 0.027, *P* = 0.002, *P* < 0.001, *P* = 0.019, respectively) (Table 3). The GERDQ scores showed a positive correlation with GERD events (*P* < 0.001). Furthermore, the results of the correlation between total GERDQ scores and the occurrence of GERD based on the Spearman's rank correlation model showed that an increasing score reflects increased risk of GERD (*r* = 0.616, *p* < 0.001). The optimal cut-off points for the GERDQ score using the receiver operating characteristic (ROC) was 1.5, and sensitivity, specificity, positive predictive value (PPV), negative predictive value (NPV), and accuracy were 75.0%, 75.0%, 77.8%, 72.0%, and 75.0%, respectively (area under the curve [AUC] 0.850; 95% CI, 0.776–0.924). We found 25 patients with GERD symptoms based on the GERDQ but with normal mucosal appearance by endoscopy, who are categorized as having NERD. In contrast, we found six patients with no symptoms of GERDQ, but who had positive mucosal breaks on endoscopy, including one patient with GERD grade C. Patients with GERD had significantly higher GERDQ scores than those with NERD (*P* < 0.001).

**Table 3:** Gastrointestinal symptoms based on GERD Questionnaire.

Symptoms		Endoscopic Finding			Total GERD (*n* = 56)	Non-GERD (*n* = 48)	*P* value*
		Grade A	Grade B	Grade C			
Heartburn	+	17	1	0	18 (32.1%)	5 (10.4%)	0.008**
	−	33	3	2	38 (67.9%)	43 (89.6%)	
Regurgitation	+	8	0	0	8 (14.3%)	1 (2.1%)	0.027**
	−	42	4	2	48 (85.7%)	47 (97.9%)	
Epigastric Pain	+	34	4	1	39 (69.6%)	18 (37.5%)	0.002**
	−	16	0	1	17 (30.4%)	30 (62.5%)	
Nausea	+	33	2	1	36 (64.3%)	8 (16.7%)	<0.001**
	−	17	2	1	20 (35.7%)	40 (83.3%)	
Sleep Disorder	+	6	0	0	6 (10.7%)	0 (0.0%)	0.019**
	−	44	4	2	50 (89.3%)	48 (100%)	
Drug Intake	+	5	0	0	5 (8.9%)	0 (0.0%)	0.105
	−	45	4	2	51 (91.1%)	48 (100%)	

*P value of GERD with symptoms vs non-GERD with symptoms.

**P < 0.05.

### Histology and GERD

On the basis of the updated Sydney system, we analyzed the histopathological scores in the antrum and corpus, and their association with GERD. There was no association between acute (neutrophil activity) and chronic gastritis (mononuclear cells) with GERD (*P* > 0.05). Among 104 biopsies, 13 (12.5%) and 2 (1.9%) patients had glandular atrophy in the antrum and corpus, respectively. There was a significant association between antral atrophic gastritis with the presence of GERD (*P* = 0.030). However, there was no significant association between corpus atrophic gastritis with GERD (*P* = 0.912) (Table 2). Moreover, when we used the OLGA score parameter, for OLGA stage 1 there was a 4.09 times higher risk of GERD than for OLGA stage 0 (*P* = 0.038). In addition, patients with GERD had significantly more severe antral atrophic gastritis than those with NERD (*P* = 0.018). Furthermore, when we excluded patients with *H*. *pylori*, the association between GERD and antral atrophic gastritis and OLGA scores was consistent (*P* = 0.025 and *P* = 0.039, respectively).

### Pepsinogen vs GERD

We could only collect 97 serum samples due to insufficient samples from the remaining seven patients. The comparison of PG levels among the patients with and without GERD is shown in Table 4. We found an association between PG I/II levels and GERD (*P* = 0.047). However, there was no association between PG I and PG II levels. When we calculated the best cut-off point using ROC for the PG I/II ratio for detecting GERD, we found the level was 6.25, and sensitivity, specificity, PPV, NPV, and accuracy were 66.0%, 59.1%, 66.0%, 59.1%, and 62.9%, respectively (AUC 0.626; 95% CI, 0.514–0.738).

**Table 4:** The comparison of pepsinogen levels between patients with and without GERD.

PG	GERD	Non-GERD	*P* value*
**PG I**			
Minimum value	14.3	16	
Maximum value	422	312	
Mean PG I ± SD	94.78 ± 77.12	78.09 ± 56.86	0.295
**PG II**			
Minimum value	2.2	3.4	
Maximum value	53.9	35.7	
Mean PG II ± SD	13.59 ± 10.32	12.17 ± 7.24	0.822
**PG I/II**			
Minimum value	3.5	4.5	
Maximum value	12	9.8	
Mean PG I/II ± SD	6.86 ± 1.68	6.23 ± 1.31	0.047**

*GERD vs non-GERD.

**Statistically significant when *P <* 0.05.

PG, pepsinogen.

### IL-1β and IL-8 polymorphism genotyping

We found three different allelic patterns based on IL-1β -511 genotyping. In total, 43 of 104 patients (41.4%) were homozygous for the wild-type allele (-511 TT), 25 of 104 patients (24.0%) were homozygous for the mutated allele (-511 CC), and 36 of 104 patients (34.6%) were heterozygous (-511 CT). On the basis of the IL-8–251 genotyping result, 37 of 104 patients (35.6%) were homozygous for the wild-type allele (-251 TT), 17 of 104 patients (16.4%) were homozygous for the mutated allele (-251 AA), and 50 of 104 patients (48.1%) were heterozygous (-251 AT).

In this genetic population study, we used the Hardy–Weinberg equation (*p*^2^ + 2*pq* + *q*^2^ = 1) to calculate the difference between observed genotype frequencies in this population and the frequencies predicted by the equation. The expected *p* value of IL-1β -511 (which is represents the T allele) was 0.59, and the expected *q* value (which represents the C allele) was 0.41. By using the same calculation, we found that the expected *p* value of IL-8–251 (which is represents T allele) was found to be 0.60 and the expected *q* value (which is represents A allele) was 0.40. Comparison of observed genotype frequencies and the predicted genotype frequencies were performed using the chi-squared test. By comparing the observed and predicted genotype frequencies, we found that genetic variants of IL-1β -511 and IL-8–251 conformed to the Hardy–Weinberg law (*χ*^*2*^ = 2.48, *P* = 0.115, and *χ*^*2*^ = 0.01, *P* = 0.920, respectively). The genotype frequencies are shown in Table 5. However, there was no statistically significant association between the IL-1β -511, IL-8–251 polymorphisms, and GERD (*P* = 0.680 and *P* = 0.242, respectively).

**Table 5:** Genotype frequencies of IL-1β -511 and IL-8–251 polymorphisms.

Genotypes	Total (%)	GERD (% within GERD)	Non-GERD (% within non-GERD)
**IL-1β -511**	104	56	48
TT	43 (41.4)	21 (37.5)	22 (45.8)
CC	25 (24.0)	14 (25.0)	11 (22.9)
CT	36 (34.6)	21 (37.5)	15 (31.3)
T carrier	79 (76.0)	42 (75.0)	37 (77.1)
C carrier	61 (58.7)	35 (62.5)	26 (54.2)
Allele T frequency	122 (58.7)	63 (56.3)	59 (61.5)
Allele C frequency	86 (41.3)	49 (43.7)	37 (38.5)
**IL-8–251**	104	56	48
TT	37 (35.6)	18 (32.1)	19 (39.6)
AA	17 (16.3)	7 (12.5)	10 (20.8)
AT	50 (48.1)	31 (55.4)	19 (39.6)
T carrier	87 (83.7)	49 (87.5)	38 (79.2)
A carrier	67 (64.4)	38 (67.9)	29 (60.4)
Allele T frequency	124 (59.6)	67 (59.8)	57 (59.4)
Allele A frequency	84 (40.4)	45 (40.2)	39 (40.6)

## Discussion

Although GERD has become one of the most common gastroduodenal disorders, its diagnosis is still a major challenge due to the absence of a gold standard for its definitive diagnosis. In this study, we have revealed that more than half of dyspeptic patients in areas with low prevalence of *H*. *pylori* were found to have GERD by endoscopy. However, more than three-quarters of these were categorized as having a mild grade of GERD which may associated with the severity of risk factors including mild atrophy of antrum. The prevalence of GERD in this report was greater than in previous studies in Indonesia. The previous study conducted in Jakarta revealed that 22.8% of the patients who had undergone upper gastrointestinal endoscopy had esophagitis [36]. Another study in Jakarta showed that the prevalence of esophagitis had increased from 5.7% in 1997 to 25.18% in 2002 [37].

The low prevalence of *H*. *pylori* among the dyspeptic patients in our study suggests that Indonesia is a good model for analyzing the controversies regarding the effect of *H*. *pylori* infection on acid reflux-related diseases. In fact, in two patients found to be *H*. *pylori*-positive, both had been diagnosed with GERD. Antral atrophic gastritis was significantly associated with non-GERD patients, suggesting that the antral atrophy might be a protective condition against GERD [38–41]. In contrast, our result showed that the higher OLGA scores had a higher risk to develop GERD. The positive association might be also attributed by the involvement of corpus atrophic gastritis. In concordance with the results, there was a significant association between PG I and PG II ratio as a biomarker of gastric mucosal status with GERD. Therefore, acid reflux-related GERD may be more important than *H*. *pylori*. Although our study proposed the best cut-off point for predicting GERD using the PG I/II ratio, the accuracy is low, suggesting that PG may not be useful for screening tests to detect patients with GERD in areas where endoscopic facilities are scarce, including Indonesia.

We found that the reflux esophagitis symptoms that were mentioned in the GERDQ, such as heartburn, regurgitation, epigastric pain, nausea, and sleep disturbance, were more frequent in the patients with GERD than in those without, with statistical significance. The total GERDQ scores also showed a positive correlation with GERD events, suggesting a benefit in distinguishing reflux esophagitis symptoms. However, mucosal breaks were found in six patients by endoscopy, without any symptoms having been mentioned in the GERDQ. Moreover, we found a low point for the optimal cut-off for GERDQ, indicating that GERDQ might not be suitable or sufficiently sensitive to distinguish GERD for the Surabaya population. These results are not surprising because the GERDQ showed wide variations in sensitivity and specificity when used in different countries, such as high sensitivity in China (87.7%) [42], but intermediate and low sensitivity in Norway and Japan (66% and 34%, respectively) [12, 43]. A previous study in Medan, a high prevalence area for *H*. *pylori* infection in Indonesia, had also shown that GERDQ was too insensitive for GERD diagnosis (49%), although with a higher cut-off value [44].

A significant association was found between GERD and smoking; the patients who smoked had a 3.60 times higher risk of developing GERD compared with nonsmokers. The risk is higher than in a previous study that found the reflux symptom risk was 1.7 times higher for smokers than for nonsmokers [15], suggesting the importance of smoking habits in the development of GERD. Smoking cessation positively improved GERD and health-related quality of life [45]; thus, it should be recommended for patients with GERD. In contrast to several studies that reported acid suppression drugs as the first-line therapy for GERD due to the inhibition of gastric acid production [46–48], we found that PPI use was positively correlated with GERD. We should note that there were differing histories of PPI intake for each patient, such as amount, dosage, duration of PPI consumption, and the time since PPI had been withdrawn, which might contribute to the result. Rebound acid hypersecretion can also occur after withdrawal of PPI therapy, thus inducing reflux-like symptoms [2, 49]. Men tended to be at higher risk of GERD than women, indicating that male patients were more likely to develop reflux esophagitis, which is consistent with previous studies [50]. The prevalence of GERD in the overweight and obesity groups also tended to be higher than in the underweight and normal groups, a result similar to several previous studies that had suggested the importance of BMI in the development of GERD [16, 17]. An association between BMI and GERD could be explained by the fact that abdominal obesity increases pressure on the stomach, consequently promoting reflux [17]. Higher economic status has been associated with BMI, suggesting that both factors could be concomitant for GERD development.

Although several studies have suggested that IL-1β -511 and IL-8–251 polymorphisms are associated with the development of various gastroduodenal disease including GERD [20, 22, 24, 51–53], no statistically significant associations have been found between IL-1β -511 and IL-8–251 polymorphisms and GERD in this study. This result might be due to the small number of samples. It is also possible that the pathogenesis of GERD does not solely depend on interleukin polymorphisms, but also on other mechanisms, such as vasoactive amines and peptides, complement components, chemokines, and hormonal regulation [54]. We used the Hardy–Weinberg equilibrium equation to determine whether the observed allele frequencies in the population differs from the predicted allele frequencies. Hardy–Weinberg law states that allele and genotype frequencies will remain constant from one generation to future generation in absence of several evolutionary influences. Equilibrium is reached in the absence of selection, mutation, and genetic drift [55]. Our results have shown that our population conformed to the law for both the IL-1β -511 and IL-8–251 polymorphisms, suggesting little disturbing factors, such as mutation, selection, gene migration, or genetic drift.

One of the limitations of this study was the relatively small sample size. In addition, we only diagnosed GERD based on endoscopy without esophageal manometry and 24-h pH monitoring, due to a lack of facilities. Other limitation is the local ethic committee allowed us to take maximum three biopsy specimens, thus we could not followed a minimum standard biopsy as Maastricht V/Florence Consensus Report which suggesting two biopsies from the antrum and the body [56]. Similar limitation also occurred to discontinue antibiotics only two weeks, but not at least 4 weeks before the test as the consensus [56]. We followed the guideline by American Society for Gastrointestinal Endoscopy for three gastric mucosal sampling [57], and used one of them for culture. Our survey showed that the detection rate of *H*. *pylori* infection using additional corporal biopsy specimens increased by 1–6% [58]. In addition, we obtained only a small number of samples from Surabaya, the eastern part of Java and the largest city in Indonesia next after Jakarta; thus, our results cannot be generalized across Java or Indonesia. To yield better corroboration of prevalence and risk factors for GERD in Indonesia, research with a larger sample size across multiple regions would be necessary.

## Conclusion

We found a high prevalence of GERD in areas with low prevalence of *H*. *pylori*; thus, GERD could instead be associated with acid reflux. The GERDQ might not be suitable or sufficiently sensitive to distinguish GERD for the Surabaya population. We have confirmed that smoking, history of PPI use, and higher economic group were significantly associated with increased risk of GERD.

## Supporting information

S1 Table GERD was diagnosed by endoscopy. NERD was defined as a condition when reflux-related symptoms are present in the absence of esophageal mucosal breaks. IL-1β and IL-8 polymorphisms were evaluated by PCR-RFLP method. *H*. *pylori* infection status was concluded by the combined result of three different tests: histology, culture, and immunohistochemistry.
(DOCX)

## References

[1] Peek RM Jr., Blaser MJ. Helicobacter pylori and gastrointestinal tract adenocarcinomas. Nature reviews Cancer. 2002;2(1):28–37. Epub 2002/03/21. 10.1038/nrc703 . 11902583 10.1038/nrc703

[2] Reimer C, Sondergaard B, Hilsted L, Bytzer P. Proton-pump inhibitor therapy induces acid-related symptoms in healthy volunteers after withdrawal of therapy. Gastroenterology. 2009;137(1):80–7, 7.e1 Epub 2009/04/14. 10.1053/j.gastro.2009.03.058 . 19362552 10.1053/j.gastro.2009.03.058

[3] Vakil N, van Zanten SV, Kahrilas P, Dent J, Jones R, Global Consensus G. The Montreal definition and classification of gastroesophageal reflux disease: a global evidence-based consensus. Am J Gastroenterol. 2006;101(8):1900–20; quiz 43. 10.1111/j.1572-0241.2006.00630.x . 16928254 10.1111/j.1572-0241.2006.00630.x

[4] el-Serag HB, Sonnenberg A. Opposing time trends of peptic ulcer and reflux disease. Gut. 1998;43(3):327–33. Epub 1998/12/24. . 9863476 10.1136/gut.43.3.327 PMC1727258

[5] Tan HJ, Goh KL. Changing epidemiology of Helicobacter pylori in Asia. Journal of digestive diseases. 2008;9(4):186–9. Epub 2008/10/31. 10.1111/j.1751-2980.2008.00344.x . 18959588 10.1111/j.1751-2980.2008.00344.x

[6] Graham DY. Helicobacter pylori update: gastric cancer, reliable therapy, and possible benefits. Gastroenterology. 2015;148(4):719–31 e3. 10.1053/j.gastro.2015.01.040 . 25655557 10.1053/j.gastro.2015.01.040 PMC4375058

[7] Lee YY, Mahendra Raj S, Graham DY. Helicobacter pylori infection—a boon or a bane: lessons from studies in a low-prevalence population. Helicobacter. 2013;18(5):338–46. 10.1111/hel.12058 . 23607896 10.1111/hel.12058 PMC3974589

[8] Qian B, Ma S, Shang L, Qian J, Zhang G. Effects of Helicobacter pylori eradication on gastroesophageal reflux disease. Helicobacter. 2011;16(4):255–65. Epub 2011/07/19. 10.1111/j.1523-5378.2011.00846.x . 21762264 10.1111/j.1523-5378.2011.00846.x

[9] Chen CL, Hsu PI. Current advances in the diagnosis and treatment of nonerosive reflux disease. Gastroenterology research and practice. 2013;2013:653989 Epub 2013/08/13. 10.1155/2013/653989 . 23935610 10.1155/2013/653989 PMC3725792

[10] Makmun D. Present status of endoscopy, therapeutic endoscopy and the endoscopy training system in Indonesia. Digestive endoscopy: official journal of the Japan Gastroenterological Endoscopy Society. 2014;26 Suppl 2:2–9. Epub 2014/04/23. 10.1111/den.12245 . 24750141 10.1111/den.12245

[11] Bai Y, Du Y, Zou D, Jin Z, Zhan X, Li ZS, et al Gastroesophageal Reflux Disease Questionnaire (GerdQ) in real-world practice: a national multicenter survey on 8065 patients. J Gastroenterol Hepatol. 2013;28(4):626–31. Epub 2013/01/11. 10.1111/jgh.12125 . 23301662 10.1111/jgh.12125

[12] Jonasson C, Wernersson B, Hoff DA, Hatlebakk JG. Validation of the GerdQ questionnaire for the diagnosis of gastro-oesophageal reflux disease. Alimentary pharmacology & therapeutics. 2013;37(5):564–72. Epub 2013/01/08. 10.1111/apt.12204 . 23289763 10.1111/apt.12204

[13] Zavala-Gonzales MA, Azamar-Jacome AA, Meixueiro-Daza A, Ramos A, J JR, Roesch-Dietlen F, et al Validation and diagnostic usefulness of gastroesophageal reflux disease questionnaire in a primary care level in Mexico. Journal of neurogastroenterology and motility. 2014;20(4):475–82. Epub 2014/10/03. 10.5056/jnm14014 . 25273118 10.5056/jnm14014 PMC4204416

[14] Eusebi LH, Ratnakumaran R, Yuan Y, Solaymani-Dodaran M, Bazzoli F, Ford AC. Global prevalence of, and risk factors for, gastro-oesophageal reflux symptoms: a meta-analysis. Gut. 2017 Epub 2017/02/25. 10.1136/gutjnl-2016-313589 . 28232473 10.1136/gutjnl-2016-313589

[15] Nilsson M, Johnsen R, Ye W, Hveem K, Lagergren J. Lifestyle related risk factors in the aetiology of gastro-oesophageal reflux. Gut. 2004;53(12):1730–5. Epub 2004/11/16. 10.1136/gut.2004.043265 . 15542505 10.1136/gut.2004.043265 PMC1774312

[16] Dore MP, Pes GM, Bassotti G, Farina MA, Marras G, Graham DY. Risk factors for erosive and non-erosive gastroesophageal reflux disease and Barrett's esophagus in Nothern Sardinia. Scand J Gastroenterol. 2016;51(11):1281–7. Epub 2016/07/07. 10.1080/00365521.2016.1200137 . 27381266 10.1080/00365521.2016.1200137

[17] El-Serag HB, Ergun GA, Pandolfino J, Fitzgerald S, Tran T, Kramer JR. Obesity increases oesophageal acid exposure. Gut. 2007;56(6):749–55. Epub 2006/11/28. 10.1136/gut.2006.100263 . 17127706 10.1136/gut.2006.100263 PMC1954847

[18] Song JH, Chung SJ, Lee JH, Kim YH, Chang DK, Son HJ, et al Relationship between gastroesophageal reflux symptoms and dietary factors in Korea. Journal of neurogastroenterology and motility. 2011;17(1):54–60. Epub 2011/03/04. 10.5056/jnm.2011.17.1.54 . 21369492 10.5056/jnm.2011.17.1.54 PMC3042219

[19] Souza RF, Huo X, Mittal V, Schuler CM, Carmack SW, Zhang HY, et al Gastroesophageal reflux might cause esophagitis through a cytokine-mediated mechanism rather than caustic acid injury. Gastroenterology. 2009;137(5):1776–84. 10.1053/j.gastro.2009.07.055 . 19660463 10.1053/j.gastro.2009.07.055

[20] Fitzgerald RC, Onwuegbusi BA, Bajaj-Elliott M, Saeed IT, Burnham WR, Farthing MJ. Diversity in the oesophageal phenotypic response to gastro-oesophageal reflux: immunological determinants. Gut. 2002;50(4):451–9. . 11889061 10.1136/gut.50.4.451 PMC1773186

[21] Cao W, Cheng L, Behar J, Fiocchi C, Biancani P, Harnett KM. Proinflammatory cytokines alter/reduce esophageal circular muscle contraction in experimental cat esophagitis. Am J Physiol Gastrointest Liver Physiol. 2004;287(6):G1131–9. 10.1152/ajpgi.00216.2004 . 15271650 10.1152/ajpgi.00216.2004

[22] Cheng HH, Chang CS, Wang HJ, Wang WC. Interleukin-1beta and -10 polymorphisms influence erosive reflux esophagitis and gastritis in Taiwanese patients. J Gastroenterol Hepatol. 2010;25(8):1443–51. 10.1111/j.1440-1746.2010.06310.x . 20659236 10.1111/j.1440-1746.2010.06310.x

[23] Taguchi A, Ohmiya N, Shirai K, Mabuchi N, Itoh A, Hirooka Y, et al Interleukin-8 promoter polymorphism increases the risk of atrophic gastritis and gastric cancer in Japan. Cancer Epidemiol Biomarkers Prev. 2005;14(11 Pt 1):2487–93. 10.1158/1055-9965.EPI-05-0326 . 16284368 10.1158/1055-9965.EPI-05-0326

[24] Yoshida N, Uchiyama K, Kuroda M, Sakuma K, Kokura S, Ichikawa H, et al Interleukin-8 expression in the esophageal mucosa of patients with gastroesophageal reflux disease. Scand J Gastroenterol. 2004;39(9):816–22. 10.1080/00365520410006729 . 15513378 10.1080/00365520410006729

[25] Indonesian Society of G. National consensus on the management of gastroesophageal reflux disease in Indonesia. Acta medica Indonesiana. 2014;46(3):263–71. . 25348191

[26] Aulia C. Prevalence of non-erosive reflux disease in Pondok Indah Hospital: a preliminary study. Acta medica Indonesiana. 2005;37(2):79–81. . 15925852

[27] Miftahussurur M, Shiota S, Suzuki R, Matsuda M, Uchida T, Kido Y, et al Identification of Helicobacter pylori infection in symptomatic patients in Surabaya, Indonesia, using five diagnostic tests. Epidemiology and infection. 2015;143(5):986–96. 10.1017/S095026881400154X . 25034254 10.1017/S095026881400154X PMC9507110

[28] Armstrong D, Bennett JR, Blum AL, Dent J, De Dombal FT, Galmiche JP, et al The endoscopic assessment of esophagitis: a progress report on observer agreement. Gastroenterology. 1996;111(1):85–92. Epub 1996/07/01. . 8698230 10.1053/gast.1996.v111.pm8698230

[29] Jones R, Junghard O, Dent J, Vakil N, Halling K, Wernersson B, et al Development of the GerdQ, a tool for the diagnosis and management of gastro-oesophageal reflux disease in primary care. Alimentary pharmacology & therapeutics. 2009;30(10):1030–8. Epub 2009/09/10. 10.1111/j.1365-2036.2009.04142.x . 19737151 10.1111/j.1365-2036.2009.04142.x

[30] Dixon M, Genta R, Yardley J, Correa P. Classification and grading of gastritis. The updated Sydney System. International Workshop on the Histopathology of Gastritis, Houston 1994. Am J Surg Pathol. 1996;20(10):1161–81. . 8827022 10.1097/00000478-199610000-00001

[31] Rugge M, Meggio A, Pennelli G, Piscioli F, Giacomelli L, De Pretis G, et al Gastritis staging in clinical practice: the OLGA staging system. Gut. 2007;56(5):631–6. 10.1136/gut.2006.106666 . 17142647 10.1136/gut.2006.106666 PMC1942143

[32] Rugge M, Genta RM, Group O. Staging gastritis: an international proposal. Gastroenterology. 2005;129(5):1807–8. 10.1053/j.gastro.2005.09.056 . 16285989 10.1053/j.gastro.2005.09.056

[33] Sugimoto M, Furuta T, Shirai N, Nakamura A, Xiao F, Kajimura M, et al Different effects of polymorphisms of tumor necrosis factor-alpha and interleukin-1 beta on development of peptic ulcer and gastric cancer. J Gastroenterol Hepatol. 2007;22(1):51–9. 10.1111/j.1440-1746.2006.04442.x . 17201881 10.1111/j.1440-1746.2006.04442.x

[34] Kang JM, Kim N, Lee DH, Park JH, Lee MK, Kim JS, et al The effects of genetic polymorphisms of IL-6, IL-8, and IL-10 on Helicobacter pylori-induced gastroduodenal diseases in Korea. J Clin Gastroenterol. 2009;43(5):420–8. 10.1097/MCG.0b013e318178d1d3 . 19077731 10.1097/MCG.0b013e318178d1d3

[35] Syam AF, Miftahussurur M, Makmun D, Nusi IA, Zain LH, Zulkhairi, et al Risk Factors and Prevalence of Helicobacter pylori in Five Largest Islands of Indonesia: A Preliminary Study. PloS one. 2015;10(11):e0140186 Epub 2015/11/26. 10.1371/journal.pone.0140186 . 26599790 10.1371/journal.pone.0140186 PMC4658100

[36] Lelosutan S, Manan C, Nur B. The Role of Gastric Acidity and Lower Esophageal Sphincter Tone on Esophagitis among Dyspeptic Patients. The Indonesian Journal of Gastroenterology, Hepatology and Digestive Endoscopy. Vol. 2(No.3):6.

[37] Syam AF, Abdullah M, Rani AA. Prevalence of reflux esophagitis, Barret's esophagus and esophageal cancer in Indonesian people evaluation by endoscopy. Cancer Research and Treatment. 2003;5(83).

[38] El-Serag HB, Sonnenberg A, Jamal MM, Inadomi JM, Crooks LA, Feddersen RM. Corpus gastritis is protective against reflux oesophagitis. Gut. 1999;45(2):181–5. Epub 1999/07/14. . 10403728 10.1136/gut.45.2.181 PMC1727606

[39] Gyawali CP. Proton Pump Inhibitors in Gastroesophageal Reflux Disease: Friend or Foe. Current gastroenterology reports. 2017;19(9):46 Epub 2017/08/07. 10.1007/s11894-017-0586-5 . 28780717 10.1007/s11894-017-0586-5

[40] Koike T, Ohara S, Sekine H, Iijima K, Kato K, Shimosegawa T, et al Helicobacter pylori infection inhibits reflux esophagitis by inducing atrophic gastritis. Am J Gastroenterol. 1999;94(12):3468–72. Epub 1999/12/22. 10.1111/j.1572-0241.1999.01593.x . 10606305 10.1111/j.1572-0241.1999.01593.x

[41] Queiroz DM, Rocha GA, Oliveira CA, Rocha AM, Santos A, Cabral MM, et al Role of corpus gastritis and cagA-positive Helicobacter pylori infection in reflux esophagitis. Journal of clinical microbiology. 2002;40(8):2849–53. Epub 2002/08/01. 10.1128/JCM.40.8.2849-2853.2002 . 12149341 10.1128/JCM.40.8.2849-2853.2002 PMC120632

[42] Wang M, Zhang JZ, Kang XJ, Li L, Huang XL, Aihemaijiang K, et al Relevance between GerdQ score and the severity of reflux esophagitis in Uygur and Han Chinese. Oncotarget. 2017;8(43):74371–7. Epub 2017/11/02. doi: 10.18632/oncotarget.20146 . 29088793 10.18632/oncotarget.20146 PMC5650348

[43] Suzuki H, Matsuzaki J, Okada S, Hirata K, Fukuhara S, Hibi T. Validation of the GerdQ questionnaire for the management of gastro-oesophageal reflux disease in Japan. United European gastroenterology journal. 2013;1(3):175–83. Epub 2014/06/12. 10.1177/2050640613485238 . 24917957 10.1177/2050640613485238 PMC4040759

[44] Siregar G, Halim S, Sitepu R. Comparison of Endoscopic Findings with Gastroesophageal Reflux Disease Questionnaires (GerdQ) and Reflux Disease Questionnaire (RDQ) for Gastroesophageal Reflux Disease in Medan. The Indonesian Journal of Gastroenterology, Hepatology and Digestive Endoscopy. 2015;Vol. 16(No. 3):5.

[45] Kohata Y, Fujiwara Y, Watanabe T, Kobayashi M, Takemoto Y, Kamata N, et al Long-Term Benefits of Smoking Cessation on Gastroesophageal Reflux Disease and Health-Related Quality of Life. PloS one. 2016;11(2):e0147860 Epub 2016/02/06. 10.1371/journal.pone.0147860 . 26845761 10.1371/journal.pone.0147860 PMC4742243

[46] Iwakiri K, Kinoshita Y, Habu Y, Oshima T, Manabe N, Fujiwara Y, et al Evidence-based clinical practice guidelines for gastroesophageal reflux disease 2015. J Gastroenterol. 2016;51(8):751–67. Epub 2016/06/22. 10.1007/s00535-016-1227-8 . 27325300 10.1007/s00535-016-1227-8

[47] Katz PO, Gerson LB, Vela MF. Guidelines for the diagnosis and management of gastroesophageal reflux disease. Am J Gastroenterol. 2013;108(3):308–28; quiz 29. Epub 2013/02/20. 10.1038/ajg.2012.444 . 23419381 10.1038/ajg.2012.444

[48] Sigterman KE, van Pinxteren B, Bonis PA, Lau J, Numans ME. Short-term treatment with proton pump inhibitors, H2-receptor antagonists and prokinetics for gastro-oesophageal reflux disease-like symptoms and endoscopy negative reflux disease. The Cochrane database of systematic reviews. 2013;(5):Cd002095 Epub 2013/06/04. 10.1002/14651858.CD002095.pub5 . 23728637 10.1002/14651858.CD002095.pub5 PMC7066537

[49] Lodrup AB, Reimer C, Bytzer P. Systematic review: symptoms of rebound acid hypersecretion following proton pump inhibitor treatment. Scand J Gastroenterol. 2013;48(5):515–22. Epub 2013/01/15. 10.3109/00365521.2012.746395 . 23311977 10.3109/00365521.2012.746395

[50] Vakil N, van Zanten SV, Kahrilas P, Dent J, Jones R. The Montreal definition and classification of gastroesophageal reflux disease: a global evidence-based consensus. Am J Gastroenterol. 2006;101(8):1900–20; quiz 43. Epub 2006/08/25. 10.1111/j.1572-0241.2006.00630.x . 16928254 10.1111/j.1572-0241.2006.00630.x

[51] Akcil G, Dogan I, Cengiz M, Engin ED, Dogan M, Unal S, et al The role of interleukin-1 gene polymorphisms and Helicobacter pylori in gastroesophageal reflux disease. Turk J Gastroenterol. 2014;25 Suppl 1:81–5. 10.5152/tjg.2014.6512 . 25910374 10.5152/tjg.2014.6512

[52] Chourasia D, Achyut BR, Tripathi S, Mittal B, Mittal RD, Ghoshal UC. Genotypic and functional roles of IL-1B and IL-1RN on the risk of gastroesophageal reflux disease: the presence of IL-1B-511*T/IL-1RN*1 (T1) haplotype may protect against the disease. Am J Gastroenterol. 2009;104(11):2704–13. 10.1038/ajg.2009.382 . 19603010 10.1038/ajg.2009.382

[53] Moons LM, Kusters JG, van Delft JH, Kuipers EJ, Gottschalk R, Geldof H, et al A pro-inflammatory genotype predisposes to Barrett's esophagus. Carcinogenesis. 2008;29(5):926–31. Epub 2008/01/15. 10.1093/carcin/bgm241 . 18192685 10.1093/carcin/bgm241

[54] Rieder F, Biancani P, Harnett K, Yerian L, Falk GW. Inflammatory mediators in gastroesophageal reflux disease: impact on esophageal motility, fibrosis, and carcinogenesis. Am J Physiol Gastrointest Liver Physiol. 2010;298(5):G571–81. 10.1152/ajpgi.00454.2009 . 20299604 10.1152/ajpgi.00454.2009 PMC2867418

[55] Namipashaki A, Razaghi-Moghadam Z, Ansari-Pour N. The Essentiality of Reporting Hardy-Weinberg Equilibrium Calculations in Population-Based Genetic Association Studies. Cell journal. 2015;17(2):187–92. Epub 2015/07/23. doi: 10.22074/cellj.2016.3711 . 26199897 10.22074/cellj.2016.3711 PMC4503832

[56] Malfertheiner P, Megraud F, O'Morain CA, Gisbert JP, Kuipers EJ, Axon AT, et al Management of Helicobacter pylori infection-the Maastricht V/Florence Consensus Report. Gut. 2017;66(1):6–30. 10.1136/gutjnl-2016-312288 . 27707777 10.1136/gutjnl-2016-312288

[57] Sharaf RN, Shergill AK, Odze RD, Krinsky ML, Fukami N, Jain R, et al Endoscopic mucosal tissue sampling. Gastrointest Endosc. 2013;78(2):216–24. Epub 2013/07/23. 10.1016/j.gie.2013.04.167 . 23867371 10.1016/j.gie.2013.04.167

[58] Miftahussurur M, Yamaoka Y. Diagnostic Methods of Helicobacter pylori Infection for Epidemiological Studies: Critical Importance of Indirect Test Validation. BioMed research international. 2016;2016:4819423 10.1155/2016/4819423 . 26904678 10.1155/2016/4819423 PMC4745376

