# Supplementary material for: Gastroesophageal reflux disease in an area with low Helicobacter pylori infection prevalence
Source: PLoS One. 2018 Nov 14;13(11):e0205644. doi: 10.1371/journal.pone.0205644 (PMC6241118; doi:10.1371/journal.pone.0205644)
Supplement: S1 Table — NERD was defined as a condition when reflux-related symptoms are present in the absence of esophageal mucosal breaks. IL-1β and IL-8 polymorphisms were evaluated by PCR-RFLP method. H. pylori infection status was concluded by the combined result of three different tests: histology, culture, and immunohistochemistry. (DOCX) [file pone.0205644.s001.docx]

**S1 Table. Patients data, interleukin genotyping, and *H. pylori* infection status.**

| **No.** | **Code** | **Sex** | **Age** | **Diagnosis** | **Genotype IL1B -511** | **Genotype IL8 -251** | ***Helicobacter pylori* Infection status** |
| --- | --- | --- | --- | --- | --- | --- | --- |
| 1 | GERD_1 | M | 34 | NERD | CT | AT | Negative |
| 2 | GERD_2 | F | 64 | NERD | CT | AT | Negative |
| 3 | GERD_3 | F | 26 | NERD | CT | TT | Negative |
| 4 | GERD_4 | M | 44 | NERD | TT | TT | Negative |
| 5 | GERD_5 | F | 27 | Non-GERD | TT | TT | Negative |
| 6 | GERD_6 | F | 52 | Non-GERD | CC | AT | Negative |
| 7 | GERD_7 | M | 46 | GERD C | CT | AT | Negative |
| 8 | GERD_8 | F | 61 | NERD | TT | TT | Negative |
| 9 | GERD_9 | M | 53 | NERD | TT | AA | Negative |
| 10 | GERD_10 | F | 48 | Non-GERD | TT | AT | Negative |
| 11 | GERD_11 | F | 37 | GERD A | CT | TT | Negative |
| 12 | GERD_12 | F | 63 | GERD A | TT | AT | Negative |
| 13 | GERD_13 | F | 22 | GERD A | CC | TT | Negative |
| 14 | GERD_14 | F | 39 | NERD | CC | AA | Negative |
| 15 | GERD_15 | M | 54 | GERD A | CT | TT | Negative |
| 16 | GERD_16 | M | 55 | NERD | TT | AT | Negative |
| 17 | GERD_17 | F | 22 | GERD A | TT | AT | Negative |
| 18 | GERD_18 | M | 47 | NERD | CC | TT | Negative |
| 19 | GERD_19 | F | 17 | NERD | TT | AT | Negative |
| 20 | GERD_20 | F | 18 | NERD | TT | AA | Negative |
| 21 | GERD_21 | F | 49 | GERD A | CT | TT | Negative |
| 22 | GERD_22 | F | 49 | NERD | CC | TT | Negative |
| 23 | GERD_23 | M | 56 | GERD A | CT | AA | Negative |
| 24 | GERD_24 | M | 32 | GERD A | TT | AT | Negative |
| 25 | GERD_25 | M | 39 | NERD | TT | AA | Negative |
| 26 | GERD_26 | F | 77 | NERD | TT | TT | Negative |
| 27 | GERD_27 | F | 59 | Non-GERD | TT | AT | Negative |
| 28 | GERD_28 | F | 28 | GERD A | CT | AT | Negative |
| 29 | GERD_29 | F | 58 | Non-GERD | CC | TT | Negative |
| 30 | GERD_30 | M | 34 | Non-GERD | CT | TT | Negative |
| 31 | GERD_31 | M | 43 | GERD A | TT | AT | Negative |
| 32 | GERD_32 | M | 45 | Non-GERD | CC | TT | Negative |
| 33 | GERD_33 | F | 24 | GERD B | TT | TT | Negative |
| 34 | GERD_34 | F | 36 | Non-GERD | CT | AA | Negative |
| 35 | GERD_35 | F | 45 | Non-GERD | CT | AA | Negative |
| 36 | GERD_36 | M | 70 | Non-GERD | TT | TT | Negative |
| 37 | GERD_37 | F | 20 | Non-GERD | CT | AT | Negative |
| 38 | GERD_38 | F | 54 | Non-GERD | TT | AA | Negative |
| 39 | GERD_39 | F | 55 | NERD | CC | AT | Negative |
| 40 | GERD_40 | F | 72 | Non-GERD | TT | TT | Negative |
| 41 | GERD_41 | F | 73 | Non-GERD | CT | AT | Negative |
| 42 | GERD_42 | F | 73 | GERD A | TT | TT | Negative |
| 43 | GERD_43 | F | 43 | GERD A | CT | AT | Negative |
| 44 | GERD_44 | F | 51 | NERD | TT | AT | Negative |
| 45 | GERD_45 | M | 66 | GERD A | CC | AT | Negative |
| 46 | GERD_46 | F | 56 | GERD A | CC | AT | Negative |
| 47 | GERD_47 | M | 29 | GERD A | TT | TT | Negative |
| 48 | GERD_48 | F | 53 | NERD | CC | AA | Negative |
| 49 | GERD_49 | M | 64 | GERD A | CT | TT | Negative |
| 50 | GERD_50 | F | 43 | GERD A | CT | TT | Negative |
| 51 | GERD_51 | F | 32 | NERD | TT | TT | Negative |
| 52 | GERD_52 | F | 38 | GERD A | CC | AT | Negative |
| 53 | GERD_53 | M | 20 | GERD A | CT | TT | Negative |
| 54 | GERD_54 | M | 63 | GERD A | CT | TT | Negative |
| 55 | GERD_55 | F | 61 | GERD A | CT | TT | Negative |
| 56 | GERD_56 | M | 64 | GERD B | CC | AT | Negative |
| 57 | GERD_57 | F | 52 | NERD | TT | AT | Negative |
| 58 | GERD_58 | M | 54 | GERD A | CT | TT | Negative |
| 59 | GERD_59 | M | 59 | GERD A | TT | TT | Negative |
| 60 | GERD_60 | F | 46 | GERD A | TT | AT | Negative |
| 61 | GERD_61 | M | 41 | Non-GERD | CT | AT | Negative |
| 62 | GERD_62 | F | 42 | GERD A | TT | TT | Negative |
| 63 | GERD_63 | M | 46 | GERD A | CT | AT | Negative |
| 64 | GERD_64 | M | 51 | GERD A | TT | AT | Negative |
| 65 | GERD_65 | M | 38 | GERD C | CC | AA | Negative |
| 66 | GERD_66 | M | 55 | Non-GERD | CT | AT | Negative |
| 67 | GERD_67 | M | 38 | GERD A | CC | AT | Negative |
| 68 | GERD_68 | M | 62 | GERD B | CT | AT | Negative |
| 69 | GERD_69 | F | 24 | GERD A | TT | AA | Negative |
| 70 | GERD_70 | F | 32 | GERD A | CC | AT | Negative |
| 71 | GERD_71 | F | 47 | GERD A | CC | AT | Negative |
| 72 | GERD_72 | M | 55 | GERD A | CC | AA | Negative |
| 73 | GERD_73 | M | 55 | GERD A | CT | AT | Positive |
| 74 | GERD_74 | F | 32 | NERD | CC | TT | Negative |
| 75 | GERD_75 | F | 61 | GERD A | CC | AT | Negative |
| 76 | GERD_76 | M | 54 | Non-GERD | TT | AT | Negative |
| 77 | GERD_77 | M | 37 | NERD | CT | TT | Negative |
| 78 | GERD_78 | F | 46 | Non-GERD | CC | TT | Negative |
| 79 | GERD_79 | F | 34 | GERD A | TT | AT | Negative |
| 80 | GERD_80 | F | 35 | GERD A | TT | TT | Negative |
| 81 | GERD_81 | F | 39 | NERD | TT | AT | Negative |
| 82 | GERD_82 | F | 39 | GERD A | CC | AT | Negative |
| 83 | GERD_83 | F | 51 | Non-GERD | CT | AT | Negative |
| 84 | GERD_84 | F | 52 | NERD | CT | TT | Negative |
| 85 | GERD_85 | M | 51 | Non-GERD | CT | TT | Negative |
| 86 | GERD_86 | M | 55 | Non-GERD | TT | AA | Negative |
| 87 | GERD_87 | F | 51 | NERD | TT | AT | Negative |
| 88 | GERD_88 | F | 57 | GERD A | TT | AT | Negative |
| 89 | GERD_89 | F | 35 | Non-GERD | CC | AA | Negative |
| 90 | GERD_90 | F | 44 | Non-GERD | CT | AT | Negative |
| 91 | GERD_91 | M | 44 | GERD A | TT | AA | Negative |
| 92 | GERD_92 | M | 57 | NERD | TT | TT | Negative |
| 93 | GERD_93 | M | 50 | GERD A | CT | AT | Negative |
| 94 | GERD_94 | M | 58 | GERD A | CT | AA | Negative |
| 95 | GERD_95 | M | 57 | GERD B | CT | TT | Negative |
| 96 | GERD_96 | M | 51 | GERD A | TT | AT | Negative |
| 97 | GERD_97 | F | 40 | GERD A | TT | AT | Negative |
| 98 | GERD_98 | M | 58 | GERD A | TT | AT | Negative |
| 99 | GERD_99 | M | 75 | GERD A | CC | AT | Negative |
| 100 | GERD_100 | F | 41 | GERD A | CT | TT | Negative |
| 101 | GERD_101 | F | 27 | GERD A | TT | AT | Negative |
| 102 | GERD_102 | M | 26 | GERD A | TT | AT | Negative |
| 103 | GERD_103 | F | 44 | GERD A | CC | AT | Negative |
| 104 | GERD_104 | F | 27 | GERD A | CT | AA | Positive |
